# Sequential allergen microarray testing during the follow-up of allergic patients

**DOI:** 10.1186/2045-7022-4-S2-P48

**Published:** 2014-03-17

**Authors:** Joana Vitte, Chantal Agabriel, Valérie Liabeuf, Isabelle Cleach, Jean-Jacques Grob, Jean-Jacques Sarles, Pierre Bongrand

**Affiliations:** 1 Marseille Medical School, Laboratoire d’Immunologie, Hôpital Conception, Marseille, France; 2 APHM Assistance Publique Hopitaux de Marseille, Service de Pédiatrie, Hôpital Timone, Marseille, France; 3 APHM Assistance Publique Hopitaux de Marseille, Service de Dermatologie, Hôpital Timone, Marseille, France; 4 APHM Assistance Publique Hopitaux de Marseille, Laboratoire d’Immunologie, Hôpital Conception, Marseille, France; 5 Marseille Medical School, Service de Dermatologie, Hôpital Timone, Marseille, France; 6 Marseille Medical School, Service de Pédiatrie, Hôpital Timone, Marseille, France

## Background

Allergen microarray testing is increasingly used as a diagnostic tool for complex allergies, but its potential as a follow-up means has not been established yet.

## Objective

To report on the specific insights brought by allergen microarray follow-up in 38 patients from Marseille (Southern France).

## Methods

From 2010 through 2013, allergen microarray testing (ISAC®, ThermoFisher Phadia) was performed more than once in 38 pediatric patients according to their clinical status, skin prick testing and previous laboratory results. A retrospective analysis of allergen microarray results is presented here.

## Results

Median age was 6 years (10 months to 17 years) when ISAC® was first performed, and 8 years (22 months to 18 years) when the latest ISAC® was performed. With respect to the number and intensity of IgE reactivities, ISAC® follow-up results entered one of the following categories: progression (16), stability (12), attenuation (8). Two patients displayed complex alterations of their molecular profile over time. In most cases, ISAC follow-up provided aid for the management of food exclusion or reintroduction regimens. Storage protein reactivity was the most frequent setting, but transition from one molecular profile to another, which were not clinically distinguishable, such as lipid tranfer proteins versus thaumatin-like proteins, was als noted. Complex cross-reactivity patterns or allergy to components which are unavailable for individual testing (sesame Ses i 1, thaumatin-like Act d 2, 7S vicillins other than those from Fabaceae, wheat Tri a 14) gained better insight from repeated ISAC assays. Finally, sequential "allergen landscape views" improved the understanding of the patient's biological and clinical evolution.

## Conclusion and clinical relevance

Sequential allergen microarray testing during the follow-up proved useful for the management and prognostic evaluation of complex polyallergic patients. Despite yielding semi-quantitative results, allergen microarray testing also helped monitoring reactivity to selected allergens such as thaumatin-like protein Act d 2. Finally, sequential allergen microarray testing provided a follow-up of each patient's progression during the atopic march, thus contributing to prognostic evaluation.

